# Predictors of Fatal Pulmonary Haemorrhage in Dogs Affected by Leptospirosis Approaching Haemodialysis

**DOI:** 10.3390/vetsci8020025

**Published:** 2021-02-08

**Authors:** Ilaria Lippi, Caterina Puccinelli, Francesca Perondi, Gianila Ceccherini, Alessio Pierini, Veronica Marchetti, Simonetta Citi

**Affiliations:** 1 Department of Veterinary Sciences, University of Pisa, Via Livornese Lato Monte, San Piero a Grado, 56122 Pisa, Italy; ilaria.lippi@unipi.it (I.L.); caterina.puccinelli@phd.unipi.it (C.P.); f.perondi87@gmail.com (F.P.); veronica.marchetti@unipi.it (V.M.); simonetta.citi@unipi.it (S.C.); 2 Ospedale Veterinario San Concordio, Via Savonarola 106/f, 55100 Lucca, Italy; gianilaceccherini@virgilio.it

**Keywords:** pulmonary haemorrhage, dog, Leptospirosis, haemodialysis, predictors

## Abstract

A retrospective case control study, which aimed to evaluate potential clinical, laboratory and imaging predictors of fatal pulmonary haemorrhage in dogs with Leptospirosis submitted to haemodialysis. The study population was divided in two groups according to the presence (PH) or absence (nPH) of pulmonary haemorrhage. A statistical comparison was performed at hospital admission for clinical (spontaneous bleeding, icterus, and respiratory distress), laboratory (serum creatinine, urea, phosphate, calcium, bicarbonate, bilirubin, AST, ALT, ALKP, GGT, total protein, albumin, glycaemia, sodium, potassium, CRP, RBC, HCT, HGB, WBC, PLT, PT, aPTT, fibrinogen), and pulmonary radiographic findings between the two groups of dogs. At hospital admission, dogs developing pulmonary haemorrhage were more likely to have respiratory distress (*p* = 0.002), severely elevated serum bilirubin (*p* = 0.002), AST (*p* = 0.04), ALT (*p* = 0.012), ALKP (*p* = 0.002), reduced serum glycaemia (*p* = 0.014), and thrombocytopenia (*p* = 0.04). Respiratory distress and elevated serum bilirubin (≥11.5 mg/dL) were independently associated with increased risk of pulmonary haemorrhage. In conclusion, the presence of respiratory distress at hospital admission is strongly associated (OR 40.9) with increased risk of pulmonary haemorrhage, even though no abnormalities are found at chest radiography.

## 1. Introduction

Severe pulmonary haemorrhagic syndrome associated to Leptospirosis was firstly described in dogs approximately ten years ago. This syndrome was characterized by a sudden and diffuse pulmonary haemorrhage, with scarce to no haemorrhagic signs in other organs [1]. In recent years, pulmonary haemorrhagic syndrome has appeared as a more and more frequent, life-threatening complication of canine Leptospirosis in some areas of Europe, while only few cases have been reported from North America. Significant differences in epidemiology of Leptospirosis, and vaccine availability between the two continents might be responsible for different clinical presentations of the disease [2,3]. Although the exact pathogenesis of pulmonary haemorrhagic syndrome is still debated, vascular damage secondary to bacterial toxins, immunologic mechanism, or disseminated intravascular coagulopathy (DIC) has been proposed as a potential pathological mechanism [4,5,6]. Histopathological lesions of dogs with pulmonary haemorrhagic syndrome have been characterized by various degrees of intra-alveolar haemorrhage. Contrary to that usually found in liver and kidneys, leptospires have been rarely identified in affected lungs [2]. In humans, pulmonary haemorrhage due to Leptospirosis is characterized by acute respiratory distress syndrome (ARDS), haemoptysis and focal pulmonary haemorrhage, with a fatality rate which can exceed 60% [7]. With the increasing evidence of an immune-mediated pathogenesis, use of immunosuppression and plasma exchange have been attempted in human patients with improvement in survival rates [8].

Historically, diagnosis of Leptospirosis has been strongly associated with a favourable outcome and prognosis in dogs submitted to intermittent haemodialysis [9]. However, recent reports from Europe showed a higher prevalence of multi-organ involvement in dogs with Leptospirosis, with a higher prevalence of severe radiological pulmonary abnormalities in non-survivors [10]. Pulmonary haemorrhage has been documented more frequently in dogs from some areas of Europe compared to the United States [11,12]. As anticoagulation in intermittent haemodialysis bases on continuous infusion of unfractionated heparin [13], dogs at risk of pulmonary haemorrhage represent a significant clinical challenge.

The aim of the present study was to retrospectively evaluate potential clinical, laboratory and imaging predictors of fatal pulmonary haemorrhage in dogs submitted to intermittent haemodialysis.

## 2. Materials and Methods

A retrospective, case-control study was performed at the Veterinary Teaching Hospital Mario Modenato of the University of Pisa. A total of 56 medical records of dogs submitted to intermittent haemodialysis with a diagnosis of Leptospirosis were reviewed. Historical (breed, age, gender, body weight, serovar of Leptospira, AKI grade, outcome, cause of death or euthanasia), clinical (urine production, spontaneous bleeding, icterus, respiratory distress), imaging (radiographic pulmonary pattern, pattern distribution, pleural effusion), and laboratory data (serum creatinine, urea, phosphate, calcium, bicarbonate, bilirubin, AST, ALT, ALKP, GGT, total protein, albumin, glycaemia, sodium, potassium, CRP, RBC, HCT, HGB, WBC, PLT, PT, aPTT, fibrinogen) were reviewed.

Diagnosis of leptospirosis was based on one or more of the following: (1) microscopic agglutination test (MAT) ≥1:800 in unvaccinated dogs, or 4-fold change in the serum titers over 1–2 weeks; (2) positive urinary and/or blood PCR. Dogs with AKI and clinical suspect of leptospirosis, which was not confirmed by MAT or PCR were excluded from the study. 

According to outcome, dogs were divided in survivors and non-survivors. Survivors (S) included dogs recovered from leptospirosis and discharged from the hospital. Non-survivors included dogs which deceased due to fatal pulmonary haemorrhage (PH), dogs which deceased or were euthanized due to pulmonary complications other than fatal pulmonary haemorrhage (P), and dogs which deceased or were euthanized due to extra-pulmonary causes (EP). Fatal pulmonary haemorrhage was defined as a sudden and dramatic clinical condition, characterized by respiratory distress and haemoptysis. Respiratory distress included clinical signs such as dyspnea, tachypnea, increased abdominal effort, and crackles and harsh lung sounds. The study population was divided in two groups according to the presence (PH) or absence (nPH) of pulmonary haemorrhage. Oliguria was defined as urine output <1 mL/k/h. 

Patients included had at least both lateral (left and right) radiographic views of the thorax at the time of the admission and they were not sedated for the radiographs. All thoracic radiographs were acquired using a high-frequency digital radiography system (MAXIVET 400 HF, Multimage s.r.l., Cavaria, Varese, Italy) and an FCR (Fuji Computed Radiography system) capsula X as image reader (FUJIFILM Corporation, Tokyo, Japan). All radiographs were reviewed by an experienced radiologist (S.C.) using a free and open-source code software program (Horos, Horosproject.org, Nimble Co LLC d/b/a Purview in Annapolis, MD, USA). The radiologist (S.C.) was unaware of the outcome for each animal. Thoracic radiographs were defined as: normal (N), unstructured interstitial pattern (UI), interstitial-alveolar pattern (IA), alveolar pattern (A). The pulmonary pattern was defined as cranial, caudal, or diffuse when involved the cranial, caudal or all lung lobes, respectively. Finally, the presence or absence of pleural fluid was reported.

All dogs included in this study underwent intermittent haemodialysis, according to their specific needs. Duration (hours), number and intensity of dialysis sessions were dependent on severity of uraemia, oliguric state, and patients’ specific requirements. According to human recommendations all AKI dogs with suspect of leptospirosis, were submitted to no-heparin haemodialysis treatments [14].

### Statistical Analysis 

Statistical data concerning continuous variables were checked for normality by Kolmogorov-Smirnov test. Normally distributed continuous variables were presented as mean ± standard deviation; abnormally distributed variables were presented as median with minimum and maximum values. Values which were normally distributed were compared between PH dogs and nPH dogs using independent-sample-*t* test. Values which were not normally distributed were compared between PH dogs and nPH dogs using Mann-Whitney *U* test. All the categorical variables were compared between PH dogs and nPH dogs using Chi-squared test. 

Binary logistic regression was used to identify continuous and categorical variables associated with the risk of pulmonary haemorrhage. Receiving operating curve (ROC) analysis was applied to continuous variables associated with a significant risk of pulmonary haemorrhage, to identify a cut off value, which showed the best combination of sensitivity and specificity. 

Data were statistically analysed with the use of SPSS (^a^IBM SPSS Statistics v. 25, IBM Corp, Redmond, WA, USA^®^). Results were considered statistically significant for *p* < 0.05.

## 3. Results

A total of 47 dogs with confirmed diagnosis of leptospirosis and submitted to intermittent haemodialysis were retrospectively included. 

Thirty dogs (64%) were males, and 17 dogs (36%) were females. Median body weight was 18 kg (5–43 kg), and median age was 3 years old (0.3–13 years). The majority of dogs were mix-breed (*n* = 30), followed by German Shepard (*n* = 3), Labrador (*n* = 3), Beagle (*n* = 2), Golden Retriever (*n* = 2), Border Collie (*n* = 2), Australian Shepard (*n* = 1), Giant Poodle (*n* = 1), Greyhound (*n* = 1), Weimaraner (*n* = 1), American Staffordshire (*n* = 1).

Thirty-nine dogs (83%) were in AKI IRIS stage 5, and 8 dogs (17%) in AKI IRIS stage 4. Thirty-four dogs (72%) showed oliguria, and 13 dogs (28%) showed normal urine production. According to diagnosis, 20 dogs were diagnosed by positive blood or urinary PCR, while 27 dogs by serum MAT (Icterohaemorrhagiae *n* = 14; Canicola *n* = 5; Pomona *n* = 6; Australis *n* = 1; Bratislava *n* = 1). Ten dogs (21%) were included in the group with pulmonary haemorrhage (PH), and 37 dogs (79%) were included in the group with no pulmonary haemorrhage (nPH). According to survival, none of the dogs of PH group survived. Pulmonary haemorrhage occurred as a sudden, and fatal event during the haemodialysis session in 8/10 dogs, while in 2/10 in the inter-dialysis period. In the nPH group, 22 dogs (59%) survived (S), 8 dogs (22%) died or were euthanized due to extra-pulmonary complications (EP), 7 dogs (19%) died or were euthanized due to pulmonary complications other than pulmonary haemorrhage (P). In EP group, 3 dogs were euthanized, and 4 died due to worsening of clinical signs of acute pancreatitis, 1 dog died of sudden cardiac arrest. In the P group, 2 dogs died, and 5 dogs were euthanized due to worsening of respiratory signs. The results of the statistical comparison of laboratory variables of the two groups are reported in Table 1. 

**Figure 1:**
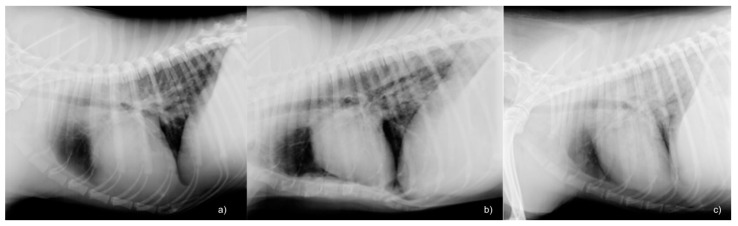
Radiographic pulmonary patterns of three dogs with radiographic abnormalities. Right lateral thoracic radiographs of three different dogs affected by leptospirosis, showing the three radiographic pulmonary patterns observed in the study; in these patients the pattern had a caudal distribution: (**a**) unstructured interstitial pattern (**b**) interstitial-alveolar pattern; (**c**) alveolar pattern.

Radiographic pulmonary abnormalities were observed in 28 dogs (59.6%). The most represented lung pattern was the unstructured interstitial (32.2%), followed by the interstitial-alveolar (28.6%) and alveolar (17.8%). Clinical and imaging findings of the different study groups are reported in Table 1. Representative examples of the three radiographic pulmonary patterns observed are reported in Figure 1.

Logistic regression showed that elevated serum bilirubin, and the presence of respiratory distress were independently associated with the risk of pulmonary haemorrhage (Table 2).

**Figure 2:**
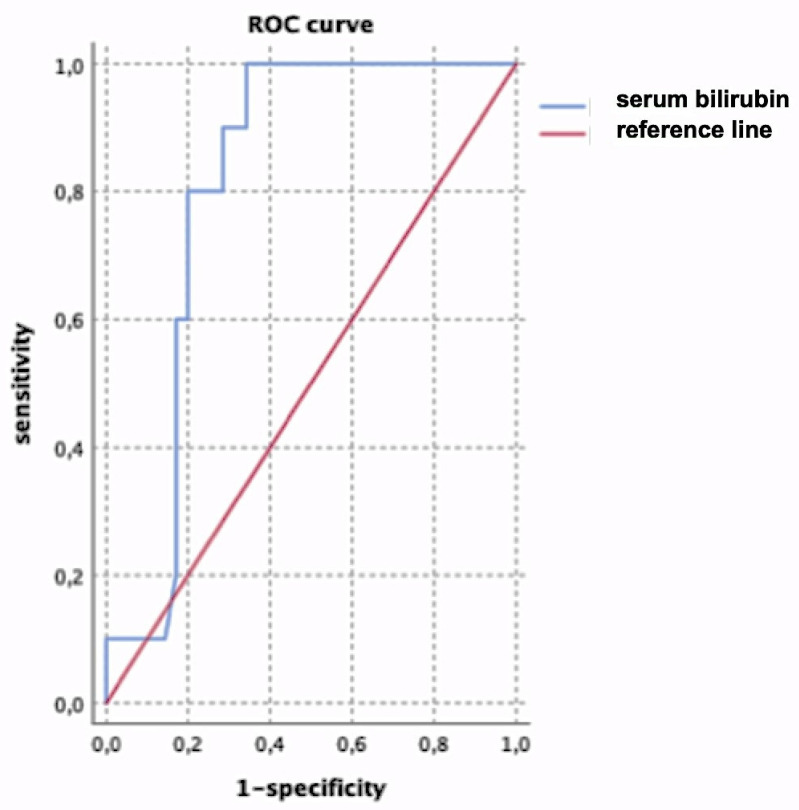
ROC curve analysis of serum bilirubin as a predictor of pulmonary haemorrhage. The best combination of sensitivity (0.8) and 1-specificity (0.2) was identified for a serum bilirubin ≥11.5 mg/dL.

ROC curve analysis showed an optimal cut-off value for serum bilirubin ≥11.5 mg/dL (0.8 of sensitivity; 0.2 of 1-specificity) (Figure 2).

PH: dogs with pulmonary haemorrhage; nPH: dogs with no pulmonary haemorrhage. Phi: Phi coefficient. Mean values of normally distributed variables were compared between the two groups through independent-sample-t test for. Median values of abnormally distributed variables were compared between the two groups through Mann Whitney U test. Categorical variables were compared between the two groups through Chi-squared test. Statistical significance was set for *p* < 0.05.

## 4. Discussion

In our study, fatal pulmonary haemorrhage occurred in 21% (*n* = 10) of dogs with a definitive diagnosis of leptospirosis. In leptospirosis dogs, which required intermittent haemodialysis, pulmonary haemorrhage contributed to 40% of overall mortality, followed by extra-pulmonary complications (32%), and pulmonary complications other than pulmonary haemorrhage (28%). In these dogs, pulmonary haemorrhage was a fatal event, characterized by massive haemoptysis and sudden death, which occurred mostly during haemodialysis. This seems to reflect previous findings in European dogs, in which pulmonary haemorrhage was a very severe clinical condition, with a mortality rate approaching 50% [15]. Although no statistically significant difference in median age was found between the two study groups, none of the enrolled puppies were present in the PH group. Our previous unpublished experiences reported an overall lower mortality rate in puppies, compared to adult dogs, when haemodialysis was used to manage AKI with leptospirosis. Although the exact pathogenesis of pulmonary haemorrhage is still debated, Schuller and colleagues reported a significant deposition of IgG and IgM in the alveoli of dogs with pulmonary haemorrhage secondary to leptospirosis [16]. Involvement of humoral immunity, and lung deposition of IgG, IgM, IgA, and C3, have been demonstrated in both experimental, and spontaneous infection [17]. Therefore, pulmonary haemorrhage may be the last step of severe AKI and acute hepatitis. As puppies between 10 and 15 weeks of age are in an “immune gap” condition, characterized by a decline of maternally derived antibodies, and low ability to produce active immunization [18], it is possible that they show a milder immune reaction, compared to adults. A similar finding was reported in human medicine, in which mean age of non-survivors was significantly higher than survivors [19].

In our cohort, respiratory distress and elevated liver enzymes at hospital admission were associated with a higher risk of pulmonary haemorrhage. In particular, respiratory distress was strongly associated with the risk of pulmonary haemorrhage. This finding is in agreement with previous reports in human medicine, where pulmonary involvement, was reported to be a strong predictor of pulmonary haemorrhage [20,21,22]. The same finding was reported for dogs affected by leptospirosis, in which non-survivors showed significantly higher prevalence of icterus and dyspnoea [10]. Similarly, the first report of an emerging pulmonary haemorrhagic syndrome, associated with leptospirosis in dogs, reported increased hepatic enzymes in all the confirmed cases of pulmonary haemorrhage [1]. Our results may strengthen the hypothesis of a critical role of concomitant hepatic and pulmonary involvement in the pathogenesis of pulmonary haemorrhage. Pulmonary haemorrhage might also be the final step of severe AKI and acute hepatitis.

Although the presence of respiratory distress was associated with higher risk of pulmonary haemorrhage, we failed to demonstrate a significant association between abnormal thoracic radiography and pulmonary haemorrhage. Radiographic pulmonary abnormalities were diagnosed in 28 cases (59.6%) at the admission. This finding was slightly greater compared to the results reported by Knopfler and colleagues (49%) [10]. Interestingly, we did not report any cases with a pulmonary reticulonodular pattern, described in leptospirosis-associated pneumopathy by previous studies. [10,11,23] One hypothesis could be that this type of pattern can be detected more frequently as the lung pathology evolves. In fact, our radiographs were performed only at the patient’s admission, unlike for example the study of Kohn and Colleagues, where for some patients, second thoracic radiographs were available, and those with more severe lung patterns were considered for the study [11]. Moreover, we reported only 6 cases of severe pulmonary pattern, considered as an interstitial-alveolar or alveolar pattern with a diffuse distribution. Also, this finding could be explained by the fact that we had available only thoracic radiographs at the patients’ admission, and we could not evaluate the possible aggravation of the pattern. Pulmonary haemorrhage was not statistically associated with a particular radiological pattern, localization, or pleural effusion. This was a very unexpected and interesting finding, as it showed that in some dogs, pulmonary haemorrhage developed as a sudden, and fatal event, within a few hours from an unremarkable thoracic radiography. A similar finding had already been reported by Knopfler and Colleagues, in which eight dogs with dyspnoea showed no abnormal thoracic radiography. However, in their study severe radiological pulmonary abnormalities were significantly more prevalent in non-survivors, compared to survivors [10]. Dogs of PH group showed a significantly lower number of PLT at hospital admission. Thrombocytopenia may be an early sign of leptospirosis, consequent to different mechanisms, such as PLT consumption due to endothelial activation, adhesion, and aggregation, immune mediated consumption, or hemophagocytosis. In human patients with severe leptospirosis, PLT < 70,000/μL was associated with a 2.6 odds ratio of lethal outcome [19]. Our findings were consistent with a previous report in dogs [1], in which thrombocytopenia was present in all dogs with pulmonary haemorrhage. However, a prospective study investigating the haemostatic status of dogs affected by leptospirosis, did not report significant difference in PLT number between survivors and non-survivors, although thrombocytopenia was more often associated with a hypocoagulable profile, and haemorrhagic diathesis [24]. In our cohort, thrombocytopenia was the only haemostasis abnormality associated with pulmonary haemorrhage. On the other side, PT, aPTT, and fibrinogen did not show to differ significantly between the study groups. Similar to what reported for coagulation profile, dogs with pulmonary haemorrhage did not show a higher tendency to bleed. This was a relevant difference compared to human patients, where people developing pulmonary haemorrhage showed significantly higher prevalence of haemorrhagic manifestations at hospital admission [25]. Unfortunately, as thromboelastography was not available, an accurate identification of hypo- and hypercoagulable state was not possible. As a consequence, the absence of spontaneous bleeding, or abnormal coagulation values in leptospirosis dog approaching haemodialysis, may not be predictive of a lower risk of pulmonary haemorrhage, or allow for the use of systemic anticoagulation.

Dogs with pulmonary haemorrhage showed significantly higher values of serum bilirubin, AST, ALT, and ALKP compared to the other groups. Previous findings by Knopfler and Colleagues, showed that non-surviving dogs with leptospirosis had higher degree of serum bilirubin and liver enzymes, apart from the cause of death [10]. Instead, in our study, both liver enzymes and bilirubin were significantly more elevated in dogs with pulmonary haemorrhage, compared to dogs which died of pulmonary or extra-pulmonary causes. In particular, serum bilirubin was reported to be an independent predictor of pulmonary haemorrhage. With the exception of one dog, all dogs of PH group showed a serum bilirubin >10 mg/dL. In particular, a serum bilirubin ≥11.5 mg/dL may be used as a predictor of pulmonary haemorrhage, with a good combination of sensitivity and specificity. Although elevated serum bilirubin and liver enzymes have been associated with a higher risk of pulmonary haemorrhage, also seen in humans with leptospirosis, the exact mechanism remains unknown [25].

This study has several limitations. Due to the retrospective nature of the study, histological evaluation of lungs was not available for any of the enrolled dogs. Diagnosis of pulmonary haemorrhage was based on a clinical evidence of severe respiratory distress and fatal haemoptysis. Therefore, it is possible that some of the dogs which died of pulmonary complications other than pulmonary haemorrhage might have had milder forms of the disease. It is also possible that overall mortality rate, and prevalence of pulmonary haemorrhage might have been overestimated, as there were dogs excluded from the study due to dubious MAT results. Although those dogs were excluded from the study, leptospirosis cannot be definitively ruled out. Thoracic radiographs were performed at time of hospital admission. Unfortunately, a daily recheck of chest radiography was not available for the majority of patients. As a consequence, no information concerning possible modifications of the radiologic pattern over time was available for review. Arterial blood gas analysis at hospital admission was available only for a very limited number of dogs. Therefore, arterial blood gas analysis could not be evaluated in the statistical analysis. C-reactive protein was available in a limited number of dogs, so we could not include this marker in the statistics. Finally, as coagulation profile did not include d-dimers and FDP, we missed information concerning a possible role of DIC in promoting pulmonary haemorrhage. 

## 5. Conclusions

In conclusion, haemodialysis dogs affected by leptospirosis, should be considered at risk of pulmonary haemorrhage in case of severe elevation of serum bilirubin (≥11.5 mg/dL), and respiratory distress. In particular, the presence of respiratory distress at hospital admission is strongly associated with increased risk of pulmonary haemorrhage, even though no abnormalities are found at chest radiography. 

## Figures and Tables

**Table 1:** Statistical comparison of laboratory, clinical and radiographic variables at hospital admission, between the two study groups.

Variables	Reference Range	PH (*n* = 10) Mean ± SD; Median (min-max)	nPH (*n* = 37) Mean ± SD; Median (min-max)	*p* Value
Creatinine	0.6–1.5 mg/dL	9.8 (8.0–22.0)	8.7 (2.3–21.1)	0.075
Urea	15–55 mg/dL	349.5 (266–611)	347 (109–626)	0.603
Calcium	8.7–11.8 mg/dL	9.6 ± 1.3	10.4 ± 2.4	0.325
Phosphate	2.5–5 mg/dL	14.4 (10.0–22.0)	13.9 (5.4–59.0)	0.795
HCO_3_	21–31 mEq/L	11.0 (6.0–16.0)	14.0 (5.0–29.0)	0.254
Bilirubin	0.07–0.3 mg/dL	17.2 ± 13.3	5.6 ± 8.5	0.002 *
AST	15–40 U/L	204.7 ± 96.0	115.6 ± 127.1	0.04 *
ALT	20–70 U/L	176.7 ± 45.9	100.7 ± 87.0	0.012 *
ALKP	45–250 U/L	1600.0 (1024.0–3541.0)	563.5 (53.0–5050.0)	0.002 *
Total Protein	5.8–7.8 g/dL	5.0 (4.0–6.8)	5.8 (4.0–9.0)	0.102
Albumin	2.6–4.1 g/dL	2.5 (2.0–2.8)	2.4 (1.3–3.7)	0.948
Glycaemia	80–125 mg/dL	83.0 (66–106)	104.5 (60–171)	0.014 *
CRP	0–0.30 mg/dL	1.6 (0.3–2.5)	1.5 (0.3–5.2)	0.557
GGT	2–11 U/L	14.7 ± 7.6	11.0 ± 10.5	0.420
Na	146–156 mEq/L	145.0 (139.0–155.0)	146.0 (135.0–163.0)	0.222
K	3.9–5.5 mEq/L	5.8 (5.0–7.0)	4.7 (2.8–8.6)	0.089
RBC	5.65–8.87 M/μL	4.8 (1.5–6.6)	4.7 (1.3–8.2)	0.761
HCT	37.3–61.7%	35 (9–39)	28 (9–53)	0.187
Hgb	13.1–20.5 g/dL	12.5 (3.5–15.0)	10.0 (4.0–19.0)	0.237
WBC	5.05–16.76 K/μL	18.0 (7.6–28.8)	18.3 (7.6–49.0)	0.720
Neutrophils	2.95–11.64 K/μL	16.5 ± 2.7	15.5 ± 6.3	0.587
Eosinophils	0.06–1.23 K/μL	0.11 ± 0.17	0.10 ± 0.13	0.862
Basophils	0–0.10 K/μL	0.03 ± 0.03	0.03 ± 0.04	0.852
Lymphocytes	1.05–5.10 K/μL	1.0 (0.1–4.8)	1.7 (0.5–8.8)	0.604
Monocytes	0.16–1.12 K/μL	2.3 (1.0–7.6)	2.0 (0.2–4.1)	0.426
PLT	148–484 K/μL	72.3 ± 47.8	225.1 ± 173.3	0.04 *
PT	5.5–11.4 sec	27.6 ± 45.4	10.6 ± 17.0	0.126
aPTT	10.6–19.9 sec	19.9 ± 2.4	21.6 ± 16.8	0.820
Fibrinogen	125–335 mg/dL	621.0 (296.0–999.0)	660.0 (103.0–2376.0)	0.688
BW	Kg	30.0 (9.0–43.0)	16.0 (5.0–43.0)	0.052
Age	Years	4.5 (2.0–10.0)	3.0 (0.3–13.0)	0.150
Icterus		8/10 (80%)	22/37 (59%)	0.230 Phi: 0.175
Spontaneous bleeding		6/10 (60%)	12/37 (32%)	0.112 Phi: 0.232
Respirtaory distress		7/10 (70%)	5/37 (13%)	0.002 * Phi: 0.457
Radiological abnormalities		6/10 (60%)	17/37 (46%)	0.177 Phi: 0.211
Radiological pattern Alveolar pattern Interstial-alveolar pattern Unstructured interstitial		2/6 (33%) 3/6 (50%) 1/6 (17%)	3/17 (18%) 5/17 (29%) 9/17 (53%)	0.225 Phi: 0.325
Radiological pattern distribution Cranial Caudal Diffuse		1/6 (17%) 1/6 (17%) 4/6 (66%)	1/17 (6%) 11/17 (65%) 5/17 (29%)	0.193 Phi: 0.340
Pleural effusion Present Absent		1/10 (10%) 9/10 (90%)	3/37 (8%) 34/37 (92%)	0.849 Phi: 0.02

* statistical significance (*p* < 0.05).

**Table 2:** Multivariate logistic regression analysis of clinical and laboratory variables associated with risk of pulmonary haemorrhage.

Variables	Logistic Regression Analysis		
	*p*	Odds Ratio	95% CI
Bilirubin	0.013 *	1.156	1.031–1.295
AST	0.380	0.993	0.978–1.009
ALT	0.324	1.016	0.985–1.048
ALKP	0.515	1.000	0.999–1.002
PLT	0.752	0.965	0.774–1.203
Glycaemia	0.172	0.926	0.828–1.034
Respiratory distress	0.003 *	40.986	3.648–460.479

OR: odds ratio; 95% CI: 95% confidence interval; R^2^ Cox and Snell: 0.424; R^2^ Nagelkerke: 0.631; * statistical significance (*p* < 0.05).

## Data Availability

Data are available in the clinical database of the Veterinary Teaching Hospital “Mario Modenato” (Department of Veterinary Sciences—University of Pisa).
